# Supplementary material for: Examination of yield, bacteriolytic activity and cold storage of linker deletion mutants based on endolysin S6_ORF93 derived from Staphylococcus giant bacteriophage S6
Source: PLoS One. 2024 Oct 23;19(10):e0310962. doi: 10.1371/journal.pone.0310962 (PMC11498662; doi:10.1371/journal.pone.0310962)
Supplement: S1 Fig — The linker protein sequences are highlighted in yellow. (A) ORF93. (B) ORF93-Δ05. (C) ORF93-Δ10. (D) ORF93-Δ15. (E) ORF93-Δ20. (PDF) [file pone.0310962.s001.pdf]

A

```
        10         20         30         40         50         60
MKTKTQALNW VNNRIGRRLD MDGAYGAQCM DLTIGYCNYL TDGKFWPWGN AKDLKSNTMP
        70         80         90        100        110        120
SGWKLIKNTP SFLPQPGDIA IWANPPYDVY GHTGIITSAN LNNFYSIDQN WFNAGSNGSP
       130        140        150        160        170        180
AAKVFHDYTG FWGVIRPAFS STATKKATTK KAAPKKKVVK KAAPKKAVTS AKWKRNSAGI
       190        200        210        220        230        240
LWKTEKATFK CNVASGIITR KNGPWTGWPT GPFMKKGDSI KYEEIQDFDG LIWVSGKFKG
       250        260        270        280        290        300
QYVYVPIGKS NGKGQRIGNA WGTFS
```

B

```
        10         20         30         40         50         60
MKTKTQALNW VNNRIGRRLD MDGAYGAQCM DLTIGYCNYL TDGKFWPWGN AKDLKSNTMP
        70         80         90        100        110        120
SGWKLIKNTP SFLPQPGDIA IWANPPYDVY GHTGIITSAN LNNFYSIDQN WFNAGSNGSP
       130        140        150        160        170        180
AAKVFHDYTG FWGVIRPAFS STATKKATTK KAVVKKAAPK KAVTSAKWKR NSAGILWKTE
       190        200        210        220        230        240
KATFKCNVAS GIITRKNGPW TGWPTGPFMK KGDSIKYEEI QDFDGLIWVS GKFKGQYVYV
       250        260        270        280        290        300
PIGKSNGKGQ RIGNAWGTFS
```

C

```
        10         20         30         40         50         60
MKTKTQALNW VNNRIGRRLD MDGAYGAQCM DLTIGYCNYL TDGKFWPWGN AKDLKSNTMP
        70         80         90        100        110        120
SGWKLIKNTP SFLPQPGDIA IWANPPYDVY GHTGIITSAN LNNFYSIDQN WFNAGSNGSP
       130        140        150        160        170        180
AAKVFHDYTG FWGVIRPAFS STATKKAVVK KAAPKKAVTS AKWKRNSAGI LWKTEKATFK
       190        200        210        220        230        240
CNVASGIITR KNGPWTGWPT GPFMKKGDSI KYEEIQDFDG LIWVSGKFKG QYVYVPIGKS
       250        260        270        280        290        300
NGKGQRIGNA WGTFS
```

D

```
        10         20         30         40         50         60
MKTKTQALNW VNNRIGRRLD MDGAYGAQCM DLTIGYCNYL TDGKFWPWGN AKDLKSNTMP
        70         80         90        100        110        120
SGWKLIKNTP SFLPQPGDIA IWANPPYDVY GHTGIITSAN LNNFYSIDQN WFNAGSNGSP
       130        140        150        160        170        180
AAKVFHDYTG FWGVIRPAFS STVVKKAAPK KAVTSAKWKR NSAGILWKTE KATFKCNVAS
       190        200        210        220        230        240
GIITRKNGPW TGWPTGPFMK KGDSIKYEEI QDFDGLIWVS GKFKGQYVYV PIGKSNGKGQ
       250        260        270        280        290        300
RIGNAWGTFS
```

E

```
        10         20         30         40         50         60
MKTKTQALNW VNNRIGRRLD MDGAYGAQCM DLTIGYCNYL TDGKFWPWGN AKDLKSNTMP
        70         80         90        100        110        120
SGWKLIKNTP SFLPQPGDIA IWANPPYDVY GHTGIITSAN LNNFYSIDQN WFNAGSNGSP
       130        140        150        160        170        180
AAKVFHDYTG FWGVIRPVVK KAAPKKAVTS AKWKRNSAGI LWKTEKATFK CNVASGIITR
       190        200        210        220        230        240
KNGPWTGWPT GPFMKKGDSI KYEEIQDFDG LIWVSGKFKG QYVYVPIGKS NGKGQRIGNA
       250        260        270        280        290        300
WGTFS
```
