# Supplementary figures and images for: Examination of yield, bacteriolytic activity and cold storage of linker deletion mutants based on endolysin S6_ORF93 derived from Staphylococcus giant bacteriophage S6
Source: PLoS One. 2024 Oct 23;19(10):e0310962. doi: 10.1371/journal.pone.0310962 (PMC11498662; doi:10.1371/journal.pone.0310962)

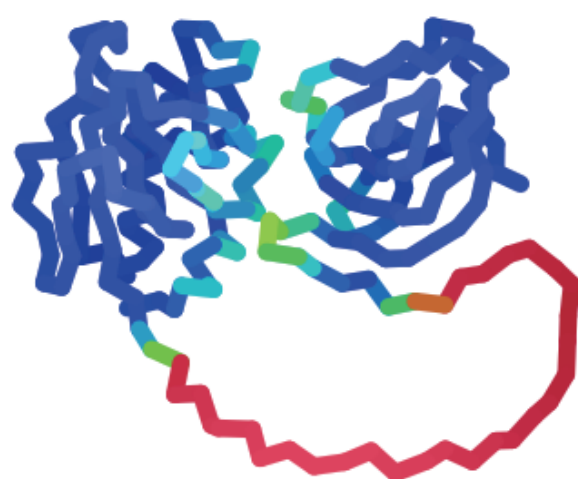

Supplement: S2 Fig — The colors on the protein structure indicate pLDDT score: < 50 in red, 50–90 by gradient color from orange, yellow, green to light blue, respectively, and > 90 in blue. The region with higher pLDDT scores indicates the structure with a higher confidence model. (PDF) [file pone.0310962.s002.pdf]

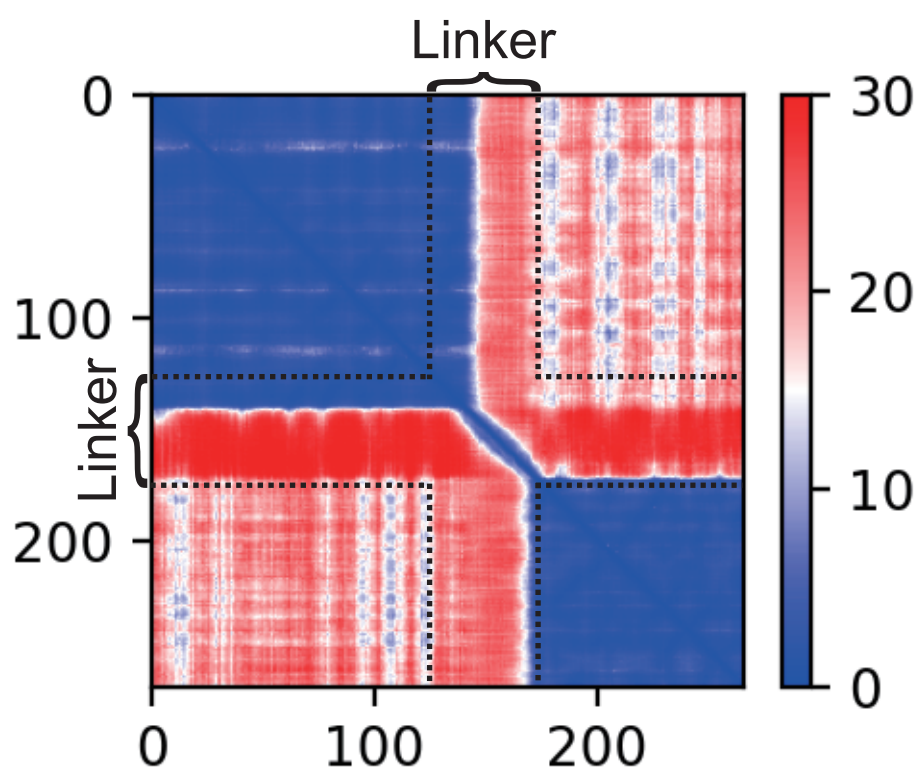

Supplement: S3 Fig — In the PAE, the scored residue x with regards to the aligned residue y is shown as a heatmap. The color scale shows the expected position error, for which the unit is Å. (PDF) [file pone.0310962.s003.pdf]

A

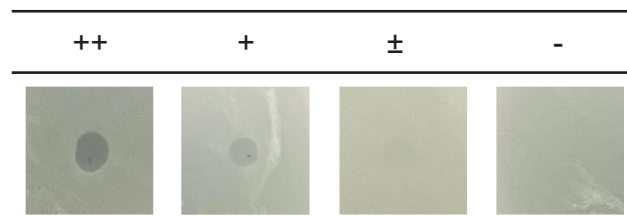

B

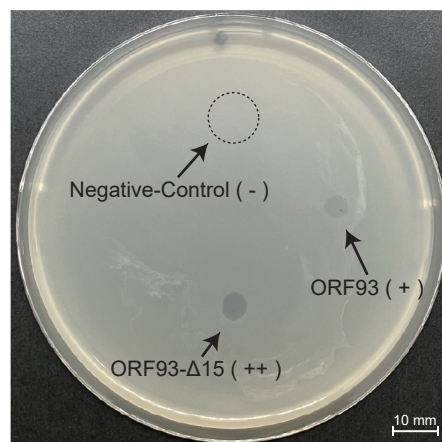

Supplement: S4 Fig — (A) Assessment criteria for the degree of the transparency of spot. The transparency of spot was evaluated at a 4-level scale: ++, strong; +, moderate; ±, weak; and -, no activity. (B) Example of antimicrobial activity examination. S. pettencoferi C58 was tested. (PDF) [file pone.0310962.s004.pdf]

(A) Original gel image presented in Fig 2A.

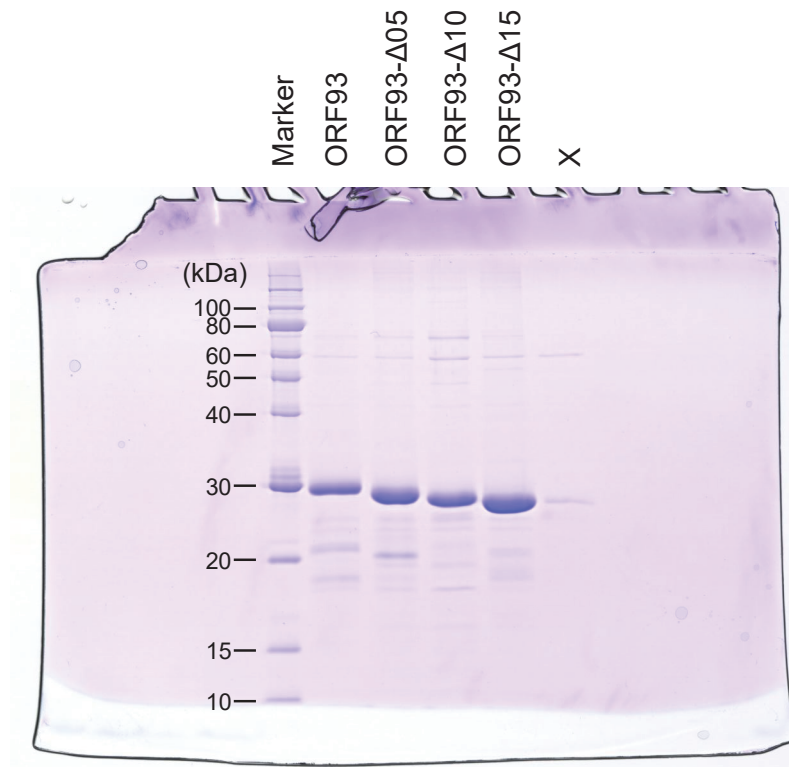

(B) Original gel image presented in Fig 4C.

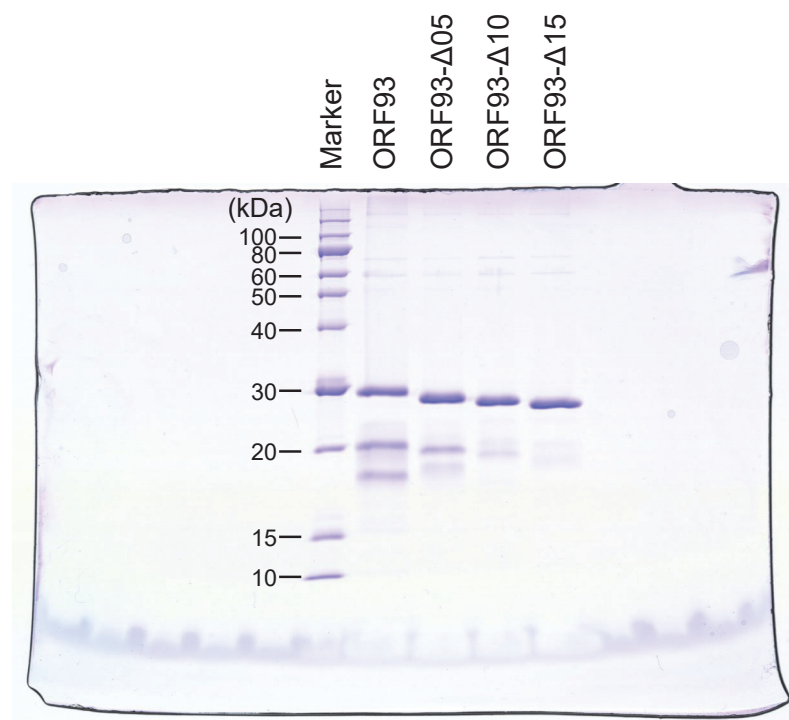

Supplement: S1 Raw images — The gel images were taken with an EPSON GT-9800F scanner (Seiko Epson Corporation, Nagano, Japan). Original gel images of Figs 2A (A) and 4C (B). (PDF) [file pone.0310962.s008.pdf]
