# Supplementary material for: Examination of yield, bacteriolytic activity and cold storage of linker deletion mutants based on endolysin S6_ORF93 derived from Staphylococcus giant bacteriophage S6
Source: PLoS One. 2024 Oct 23;19(10):e0310962. doi: 10.1371/journal.pone.0310962 (PMC11498662; doi:10.1371/journal.pone.0310962)
Supplement: S5 Fig — In this study, we calculated the bacteriolytic activity from the above equation using the OD595 value at 30 min. (PDF) [file pone.0310962.s005.pdf]

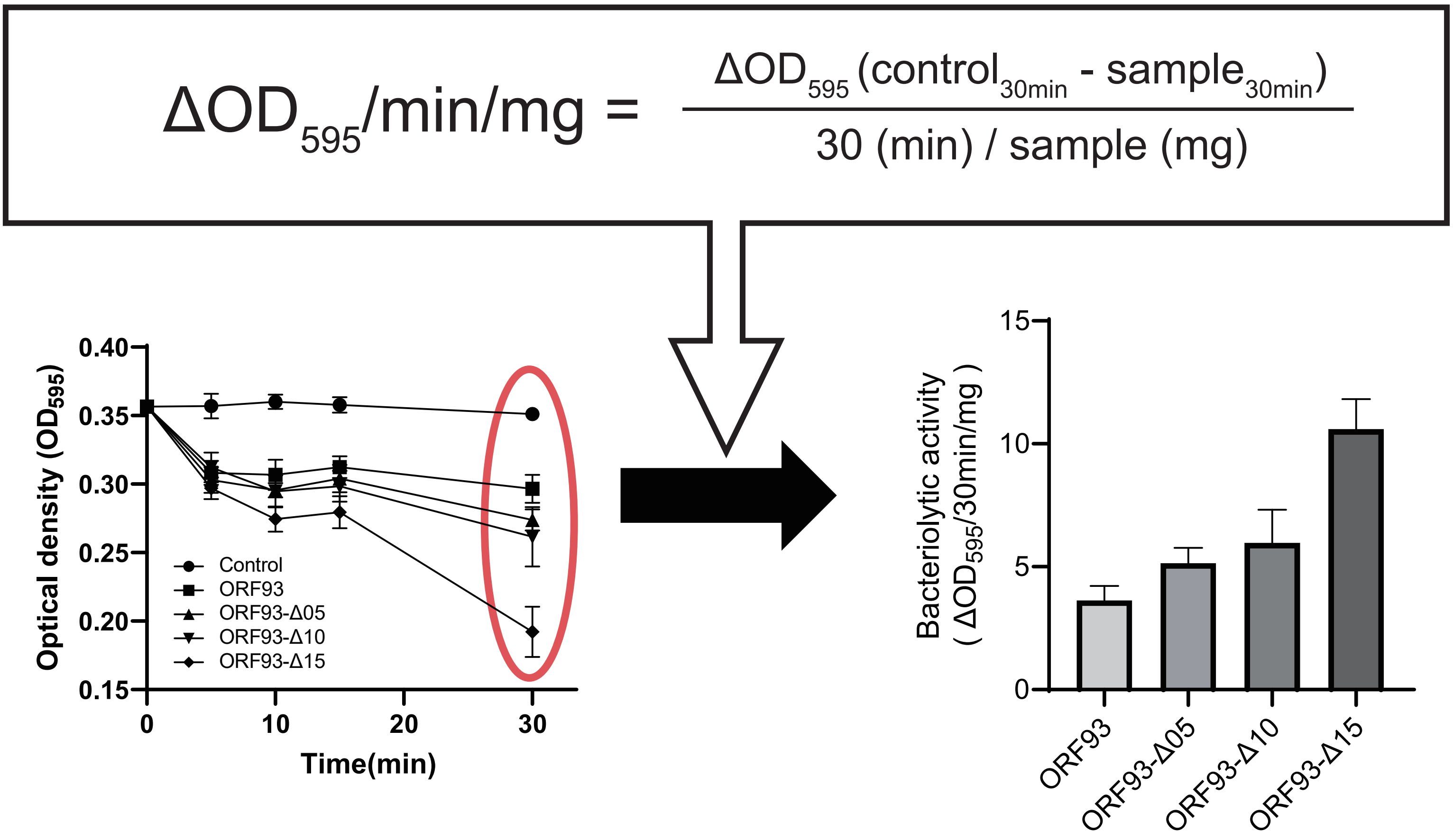

ΔOD595/min/mg = ΔOD595 (control30min - sample30min) / 30 (min) / sample (mg)
Optical density (OD595)
0.40
0.35
0.30
0.25
0.20
0.15
0
10
20
30
Time(min)
Control
ORF93
ORF93-Δ05
ORF93-Δ10
ORF93-Δ15
Bacteriolytic activity
(ΔOD595/30min/mg)
15
10
5
0
ORF93
ORF93-Δ05
ORF93-Δ10
ORF93-Δ15
