# Supplementary material for: Examination of yield, bacteriolytic activity and cold storage of linker deletion mutants based on endolysin S6_ORF93 derived from Staphylococcus giant bacteriophage S6
Source: PLoS One. 2024 Oct 23;19(10):e0310962. doi: 10.1371/journal.pone.0310962 (PMC11498662; doi:10.1371/journal.pone.0310962)
Supplement: S6 Fig — The linker DNA sequence is highlighted in yellow. (PDF) [file pone.0310962.s006.pdf]

The 15 bp sequence complement to the linearized pUC19 vector

*Sac* I

| 10 | 20 | 30 | 40 | 50 | 60 |
|---|---|---|---|---|---|
| CGGTACCCGG | GGATCGAGCT | CAAGACCAAA | ACACAAGCTC | TTAACTGGGT | GAATAACCGT |
| 70 | 80 | 90 | 100 | 110 | 120 |
| ATTGGCCGGC | GTTTGGATAT | GGATGGTGCC | TACGGTGCCC | AATGTATGGA | TCTCACGATC |
| 130 | 140 | 150 | 160 | 170 | 180 |
| GGCTACTGCA | ATTATCTTAC | GGACGGGAAG | TTCTGGCCTT | GGGGTAATGC | TAAGGATCTC |
| 190 | 200 | 210 | 220 | 230 | 240 |
| AAATCCAATA | CTATGCCGAG | TGGGTGGAAA | TTGATTAAAA | ACACGCCGTC | GTTTTTGCCA |
| 250 | 260 | 270 | 280 | 290 | 300 |
| CAGCCGGGTG | ACATTGCCAT | CTGGGCCAAT | CCTCCATACG | ACGTGTATGG | GCATACAGGG |
| 310 | 320 | 330 | 340 | 350 | 360 |
| ATTATTACTT | CCGCCAACCT | GAACAATTTT | TATAGTATTG | ACCAAAATTG | GTTTAATGCG |
| 370 | 380 | 390 | 400 | 410 | 420 |
| GGTTCTAACG | GGAGTCCTGC | TGCCAAGGTA | TTCCACGACT | ACACCGGTTT | CTGGGGTGTC |
| 430 | 440 | 450 | 460 | 470 | 480 |
| ATTCGGCCTG | CGTTTTCGAG | CACAGCGACC | AAAAAAGCTA | CTACCAAGAA | GGCTGCCCCA |
| 490 | 500 | 510 | 520 | 530 | 540 |
| AAGAAGAAAG | TTGTCAAAAA | GGCTGCACCG | AAGAAGGCTG | TAACATCAGC | TAAGTGGAAA |
| 550 | 560 | 570 | 580 | 590 | 600 |
| CGTAATAGTG | CTGGGATTCT | GTGGAAGACG | GAGAAGGCGA | CCTTCAAGTG | TAATGTTGCG |
| 610 | 620 | 630 | 640 | 650 | 660 |
| TCTGGTATTA | TTACGCGTAA | GAATGGTCCG | TGGACGGGTT | GGCCTACAGG | CCCGTTCATG |
| 670 | 680 | 690 | 700 | 710 | 720 |
| AAAAAGGGCG | ATTCGATTAA | GTACGAAGAA | ATTCAGGATT | TTGACGGCCT | TATTTGGGTT |
| 730 | 740 | 750 | 760 | 770 | 780 |
| TCTGGGAAAT | TTAAAGGTCA | ATACGTGTAC | GTTCCGATCG | GGAAATCCAA | TGGGAAGGGC |
| 790 | 800 | 810 | 820 | 830 | 840 |
| CAACGGATTG | GCAACGCCTG | GGGTACTTTC | AGCTCTAGAG | ATCCTCTAGA | GTCG |

*Xba* I

The 15 bp sequence complement to the linearized pUC19 vector
