# Supplementary material for: Examination of yield, bacteriolytic activity and cold storage of linker deletion mutants based on endolysin S6_ORF93 derived from Staphylococcus giant bacteriophage S6
Source: PLoS One. 2024 Oct 23;19(10):e0310962. doi: 10.1371/journal.pone.0310962 (PMC11498662; doi:10.1371/journal.pone.0310962)
Supplement: S1 Table — (DOCX) [file pone.0310962.s007.docx]

**Supplementary Table S1**. List of the PCR primers used in this study.

| **Primer** | **Sequence (5’ to 3’)** | **Note** |
| --- | --- | --- |
| d05F | ACTACCAAGAAGGCTGTTGTCAAAAAGGCTGCACCGAAGAAG | Used to produce the *orf93-Δ05* gene |
| d05R | AGCCTTCTTGGTAGTAGCTTTTTTGGTCG |  |
| d10F | GCGACCAAAAAAGCTGTTGTCAAAAAGGCTGCACCGAAGAAG | Used to produce the *orf93-Δ10* gene |
| d10R | AGCTTTTTTGGTCGCTGTGCTCGAAAAC |  |
| d15F | GCGTTTTCGAGCACAGTTGTCAAAAAGGCTGCACCGAAGAAG | Used to produce the *orf93-Δ15* gene |
| d15R | TGTGCTCGAAAACGCAGGCCGAATG |  |
| d20F | GGTGTCATTCGGCCTGTTGTCAAAAAGGCTGCACCGAAGAAG | Used to produce the *orf93-Δ20* gene |
| d20R | AGGCCGAATGACACCCCAGAAACC |  |
