# Supplementary material for: Examination of yield, bacteriolytic activity and cold storage of linker deletion mutants based on endolysin S6_ORF93 derived from Staphylococcus giant bacteriophage S6
Source: PLoS One. 2024 Oct 23;19(10):e0310962. doi: 10.1371/journal.pone.0310962 (PMC11498662; doi:10.1371/journal.pone.0310962)
Supplement: Attachment — Submitted filename: Response to Reviewers.docx [file pone.0310962.s009.docx]

We thank the Editor and the Reviewers for their constructive and insightful comments.

**Responses to the Editor’s comments**

The Editor made 8 comments. We have responded to them all and have revised the manuscript accordingly.

1. The Editor pointed out that the manuscript does not follow the PLOS ONE style. We have checked this and modified it. However, we did not find any errors with regard to naming the files.

**Manuscript**: The manuscript is now double-spaced.

**Line 1:**“Running title: Examination … linker deletion endolysin” has been deleted.

**Lines 3–5:** The title has been changed from left-aligned to centered.

2. The Editor pointed out that the manuscript had overlaps with previous work. Because we considered the possibilities of plagiarism, we used plagiarism detection software iThenticate (Turnitin, US, <https://www.turnitin.com/>) (checked on 07/24/2024). This software did not detect any evidence of plagiarism. We have attached the iThenticate outcome in this revision. We are not certain if the Editor’s comment in this regard is correct. If our response is not an appropriate one, please specify the lines and/or sections of overlapping text so that we can further revise the manuscript.

Moreover, the Editor suggested that we should address the way in which our manuscript constituted advances compared with previous work. We agree with this comment and have thus modified the manuscript as follows.

**Line 324:** The following sentence has been added.

“This study addresses the subject more comprehensively than previous articles and shows that optimizing linker length could have multiple benefits for endolysin development.”

3. The Editor noted that we should state the role of the study’s funder in the cover letter. In the cover letter, we state, “The funders had no role in the study design, data collection and analysis, decision to publish, or preparation of the manuscript.”
4. The Editor noted that the funding information should be removed from the manuscript and added to the cover letter. We have therefore removed “This work was funded by … Zenyaku Kogyo Co. Ltd.” in line 419 of the manuscript. In the cover letter, we have stated “This work was funded by a joint research fund from Nippon Zenyaku Kogyo Co. Ltd. (<https://www.zenoaq.com/>). J.U. has received this funding. There is no specific grant number.”
5. The Editor noted that the statement about competing interests should be included in the cover letter. We have inserted the sentence “The authors declare that no competing interests exist.”
6. The Editor required us to provide the original raw images for all the figures as supplementary data. We have added the document S1_raw_images.pdf in the Supporting information. Because of this modification, the following sentences have been added in line 670.

**S1_raw_images. Raw data of SDS-PAGE gel stained with Coomassie Brilliant Blue R250.** The gel images were taken with an EPSON GT-9800F scanner (Seiko Epson Corporation, Nagano, Japan). Original gel images of Fig 2A (A) and Fig 4C (B).

We also noted in the cover letter that the original images for gel data were provided in the Supporting information.

7. The Editor commented that our Supporting information file [S1-S6_Fig] could not be opened. We apologize for this inconvenience. We have amended this issue by modifying file format from EPS to PDF format.
8. The Editor requested us to review the reference list. We have reviewed the reference list. We have adjusted the reference list to comply with the PLOS ONE style. We have not found any retracted articles in the reference list. If there are any, please state those concerned.

**Responses to Reviewer #1’s comments**

We have responded to one general comment and three specific ones and revised the manuscript accordingly.

**Response to the general comment**

1. Reviewer #1 recommended including some references supporting the fact that the optimal linker is crucial to the protein function. We have revised the manuscript to strengthen our manuscript as follows, with the addition of the suggested references.

**Line 82:** “The influence of linker … been examined well, to our knowledge.” has been replaced with the following sentences.

“For example, the expression of protein without a linker was not successful in *Escherichia coli* [20]. Changing the codons of the linker improved the expression of fusion protein [21]. The linker length is shown to be an important factor for protein characteristics such as stability [22], activity [23], and catalytic efficiency [24]. However, the influence of linker length on the endolysin characteristics has not been examined well, to our knowledge.”

**Line 88:** “*Escherichia coli*” has been replaced with “*E. coli*.”

**Response to specific comments**

2. Reviewer #1 suggested that “amino acid residues” be replaced with “amino acids.” We agree with this suggestion and have made this change throughout the text.

**Lines 94, 95, 99–100, 106, 111, 115, 122, and 138:** “amino acid residue” has been replaced with “amino acid”.

**Lines 35, 100–101, 109, 111, 122, and 264:** “amino acid residues” has been replaced with “amino acids”.

3. Reviewer #1 requested us to mention the software used to determine the molecular masses of studied proteins. We have modified the manuscript as follows.

**Line 383:** The following sentence has been added.

“The theoretical molecular mass was calculated from the protein sequence using GENETYX^®^ Ver. 14.1.1 (Nihon server, Tokyo, Japan); the protein molecular mass was estimated from the thickest band in the SDS-PAGE image using ImageJ software (version 1.54g) [58].”

4. Reviewer #1 requested us to mention the protein quantity used in bacteriolytic activity tests in line 184. We have modified the manuscript as follows.

**Line 184:** “In the assay, the endolysin … examined after incubation.” has been replaced with the following sentences.

“In the assay, the endolysin solutions (3 µL of 0.1 mg/mL) were applied on the surface of double-layered culture media containing bacteria, and the transparency of the spot formed on the agar plate was examined after incubation.”

**Responses to Reviewer #2’s comments**

Reviewer #2 made 4 comments. We have responded to these comments and revised the manuscript accordingly.

1. Reviewer #2 suggested merging Figs 1 and 2. We have done so, and this is now included as a new Fig 1. We have modified the manuscript as follows.

**Line 114:** “**Fig 1. Description of ORF93.** (A) Schematic … correspond to Fig 1A.” has been replaced with the following sentences.

“**Fig 1. Description of ORF93 and its mutants.** The domains and linker are colored as follows: CHAP domain in green, linker in red, and SH3 domain in yellow. (A) Schematic diagram of ORF93 based on the primary structure. Each number represents the amino acid position from the N-terminus at both ends of the domain, counted from the N-terminus. (B) Predicted protein structure of ORF93. (C) Schematic diagram of ORF93 mutants based on the primary structure. The linker deletions are shown as shadowed areas. The numbers around shadowed areas represent the amino acid position from the N-terminus at both ends of the deletion area. ORF93-Δ05, ORF93-Δ10, ORF93-Δ15, and ORF93-Δ20 are the ORF93 mutants which 5, 10, 15, and 20 amino acids were deleted in the upstream direction from the 157^th^ amino acid from the N-terminus. The predicted linker lengths are 21, 16, 11, and 6 amino acids, respectively. (D) Predicted protein structures of ORF93-Δ05, ORF93-Δ10 and ORF93-Δ15. These protein structures were overlapped based on the CHAP domain. (E) Predicted protein structure of ORF93-Δ20.”

**Line 121:** “Fig 2A” has been replaced with “Fig 1C”.

**Line 127:** “Fig 2B” has been replaced with “Fig 1D”.

**Line 129:** “Fig 2C” has been replaced with “Fig 1E”.

**Line 132:** “**Fig 2. Description of ORF93 mutants.** The domains and linker … protein structure of ORF93-Δ20.” has been deleted.

**Line 151:** “Fig 3A” has been replaced with “Fig 2A”.

**Line 160:** “Fig 3” has been replaced with “Fig 2”.

**Line 175:** “Fig 3B” has been replaced with “Fig 2B”.

**Line 214:** “Fig 4” has been replaced with “Fig 3”.

**Line 222:** “Figure 4” has been replaced with “Fig 3”.

**Line 232:** “Fig 5A” has been replaced with “Fig 4A”.

**Line 236:** “Fig 5B” has been replaced with “Fig 4B”.

**Line 241:** “Fig 5C” has been replaced with “Fig 4C”.

**Line 243:** “Fig 5” has been replaced with “Fig 4”

2. Reviewer #2 addressed the stability of the proteins at room temperature (i.e., how long can the modified protein maintain activity at room temperature?). We appreciate this comment. The storability can extend the usability of endolysins. We consider this information to be highly appropriate when discussing general endolysin characteristics. Thus, we have modified the manuscript as follows.

**Line 322: A new paragraph has been added as follows.**

“To study the feasibility of endolysins, they must be investigated from other aspects, such as physical characteristics and therapeutic effects in vivo. First, physical characteristics such as pH stability and thermostability are important to develop an endolysin drug, because protein instability leads to irreversible aggregation and is a major concern in the development of new biopharmaceuticals. In particular, these characteristics have been investigated in detail to develop monoclonal antibodies as biopharmaceuticals [52, 53]. Similarly to this, the physical characteristics of endolysins must be investigated regarding their suitability in biopharmaceuticals in the future. In addition, the additives enabling the protein stability are also important, to extend the usability to an endolysin drug. For example, a previous study has shown that the activity of LysK endolysin was maintained at 100% when stored at 22 °C for one month with polyols such as glycerol, whereas without polyols, it decreased to 5% [54]. Moreover, the therapeutic effects in vivo may be varied depending on endolysin type. In the previous study, some endolysins have shown therapeutic effects equivalent to vancomycin in mice with systemic MRSA infections, whereas other endolysins have not [12]. In addition, it has been shown that endolysins exhibit different levels of biofilm removal activity [12]. We need to investigate the therapeutic effects in vivo with an understanding of the biochemical properties of endolysins.”

3. Reviewer #2 recommended conducting further experiments, such as temperature stability, pH stability tests, biofilm removal tests, in vivo antibacterial tests, etc. We appreciate these comments. However, our intention is to conduct these experiments in the future, because of the limits to the manuscript length and thus its content. Instead, we have reviewed the work on endolysins again, and have added these points to the new paragraph described right above in the response to comment No. 2 of Reviewer #2.
4. Reviewer #2 raised questions on the effects of extending the linker, and of altering or optimizing the linker’s amino acid sequence on endolysin function. We have modified the manuscript as follows.

**Lines 317–321:** “Moreover, phage S6 … to maximize endolysin function [14-16, 32, 33, 40, 47]” has been replaced with “In addition, phage S6 is a giant phage, and a longer linker of ORF93 may produce larger holes in the peptidoglycan by simply extending the distance from CHAP to the SH3 domain.”

**Line 321: A new paragraph has been added as follows.**

“To understand the role of the linker further, it is also important to examine both linker extension and modification. A previous study showed that a longer linker improves the thermostability of subunit-fused nitrile hydratase; however, an excessively long linker can decrease its activity and expression [47], which is consistent with the results of this study. Moreover, in protein engineering, various linkers have been designed and classified as flexible, rigid, helical, and cleavable linkers [19, 49-51]. It has been reported that changing the type of linker can change the stability and activity of the enzyme [38, 47, 48]. In the future, the linker types will be examined in ORF93-Δ15.”

**Minor modifications**

Through this revision, we have found minor mistakes in the abstract. Thus, we have modified our manuscript as follows.

**Line 32:** "linker modification" has been replaced with "linker shortening".

**Line 36:** "all the endolysins" has been replaced with "ORF93 and its mutants".

**Line 51:** “(VISA)” has been deleted.

**Line 52:** “(VRSA)” has been deleted.

**Lines 658–659:** “The vertical and horizontal … of ORF93, respectively” has been replaced with “In the PAE, the scored residue *x* with regards to the aligned residue *y* is shown as a heatmap”.
